# Promoting Influenza Vaccination among Staff of Nursing Homes According to Behavioral Insights: Analyzing the Choice Architecture during a Nudge-Based Intervention

**DOI:** 10.3390/vaccines8040600

**Published:** 2020-10-12

**Authors:** Chiara Lorini, Francesca Ierardi, Claudia Gatteschi, Giacomo Galletti, Francesca Collini, Laura Peracca, Patrizio Zanobini, Fabrizio Gemmi, Guglielmo Bonaccorsi

**Affiliations:** 1 Department of Health Science, University of Florence, 50134 Florence, Italy; patrizio.zanobini@unifi.it (P.Z.); guglielmo.bonaccorsi@unifi.it (G.B.); 2 Quality and Equity Unit, Regional Health Agency of Tuscany, 50141 Florence, Italy; francesca.ierardi@ars.toscana.it (F.I.); claudia.gatteschi@ars.toscana.it (C.G.); giacomo.galletti@ars.toscana.it (G.G.); francesca.collini@ars.toscana.it (F.C.); peraccal@gmail.com (L.P.); fabrizio.gemmi@ars.toscana.it (F.G.)

**Keywords:** influenza vaccination, nursing home, choice architecture, nudge, staff

## Abstract

(1) Background: Influenza vaccination uptake in nursing home (NH) workers is uncommon. The aim of this study was to understand the choice architecture of influenza vaccination acceptance or refusal among them and to promote vaccination acceptance using the nudge approach. (2) Methods: In autumn 2019, a nudge intervention with a contextual qualitative analysis of choice architecture of vaccination was performed among the staff of eight Tuscan NHs. In summer 2020, a cross-sectional study including the staff of 111 NHs (8 in the nudge, 103 in the comparison group) was conducted to assess the impact of the nudge intervention in promoting vaccination uptake. (3) Results: Macro-categories of motivations for vaccination uptake that emerged from the qualitative analysis were risk perception, value dimension, and trust, while those regarding refusal were risk perception, distrust, value dimension, and reasons related to one’s health. Considering the cross-sectional study, influenza vaccination uptake in the 2018–2019 season was similar in the two groups (23.6% vs. 22.2% respectively, in the nudge and comparison group), but significantly different in the 2019–2020 season: 28% in the nudge vs. 20% in the comparison group. Also, the intention to get the vaccine in the 2020–2021 season was significantly different in the two groups: 37.9% in the nudge and 30.8% in the comparison group. (4) Conclusions: Nudge interventions-simple, fast, low cost-could be effective in promoting vaccination acceptance among NH workers and the analysis of choice architecture could be useful in improving tailored, new nudge interventions aimed at modifying irrational biased and cognitive errors.

## 1. Introduction

Influenza is a highly contagious viral disease, that affects about 5–15% of the population worldwide and contributes to a substantial annual burden of deaths globally [1,2]. Illnesses range from mild to severe and even death, but hospitalization and death occur mainly among high-risk groups [3]. One of the most vulnerable groups for severe disease and influenza-related complications are older people; globally, 67% of the deaths occurred among people aged 65 years and older and the widest range of influenza-associated mortality rates was observed among people aged 75 years and more [2,3]. Nursing home (NH) environments and the vulnerability of their residents provide a setting that allows the rapid spread of influenza. Outbreaks in such settings, related to the introduction of influenza virus by staff, visitors, or new residents, can lead to heavy consequences for older people’s health, and for the healthcare services provided in those facilities (due to absenteeism of the sick staff and the associated costs) [4].

Influenza vaccination is effective in reducing the burden of influenza illness among older people in NHs [5,6,7], although the immunosenescence which accompanies aging may limit their protection [8]. For this reason, vaccination of the NHs staff that give assistance to older people is required in order to contribute in reducing the outbreaks and their burden [8]. Nonetheless, influenza vaccination uptake among staff in NHs is generally low [9,10,11,12]. Reasons for such a low influenza vaccination uptake may include a wide range of factors that can be encompassed within the phenomenon of vaccine hesitancy, that refers to “delay in acceptance or refusal of vaccination despite availability of vaccination services” [13]. Interventions aimed at promoting vaccination uptake among the staff of NHs could be devoted to improving access to vaccination, eliminate individual barriers, or introduce policy interventions [14]. This could be particularly crucial in some geographical contexts such as Italy, where: (i) influenza vaccination for healthcare workers and, in general, for staff members of NHs is not mandatory (but strongly recommended), (ii) no compensation for getting the vaccine is provided (but influenza vaccination is free of charge for such target groups), and (iii) vaccination is rarely provided to staff members at the NHs where they work (they generally have to contact their general practitioner to get the vaccine).

Behavioral science theory could help both in understanding the reasons for vaccination acceptance or refusal, and in promoting vaccination uptake through “nudges”.

Nudge-based interventions lean on behavioral science theories, they work by assuming that the “choice architecture” on the background of the individual is often based on biased choices [15], that can be modified in order to facilitate the adoption of an appropriate and socially desirable behavior, without limiting the options that can be chosen [15].

Many people are already quite familiar with nudges, since they are frequently adopted in shape marketing strategies: we usually experience “nudges” when we are booking plane tickets online, and the airlines website requires us to actively choose whether or not to purchase the trip insurance before we get to the buying option, or when we are suggested to order additional and complementary items on commercial websites, or when a web TV default options automatically address to play the next episode in a TV series. Shifting to a nudge approach example in a healthcare setting, physicians can be nudged to prescribe generically rather than brand-name formulated medication, simply by setting in the electronic health record the default option for the former and requiring an opt out choice for the latter [16].

Since nudge interventions appeared to be effective in different healthcare settings [16], in 2019, the World Health Organization (WHO) published a manual [17] for national immunization program managers and policy makers that includes nudge interventions among the ones that can promote the vaccination uptake in healthcare workers. Guided choice across default options and increasing options to obtain vaccination appear the most effective nudges within an intervention toolkit that includes also the pre-commitment to obtain vaccination, the information framing with social comparison feedback, and the information on one’s own and others’ benefits [18,19].

A recent research conducted in Tuscany (Italy) in 28 NHs revealed that only about 16.6% of the staff was vaccinated against influenza in 2017–2018, and 28.4% declared the intention to get the vaccine in 2018–2019; moreover, vaccine confidence was the strongest predictor of vaccine acceptance [12]. Considering these results, a new study was conducted in a sample of Tuscan NHs to understand the choice architecture of influenza vaccination acceptance or refusal among the staff of nursing homes and to promote vaccination acceptance using the nudge approach.

## 2. Materials and Methods

### 2.1. Study Design

A nudge intervention with a contextual analysis of choice architecture of vaccination acceptance or refusal was proposed to the Chief Officers of 28 Tuscan NHs that had participated in a previous study, conducted in 2018, with the aim of investigating influenza vaccination acceptance among staff of NHs, as described in a recent publication [12]. Eight of them voluntary joined the study. In those NHs, in November 2019, a letter drawn up using the nudge approach (intervention) and an anonymous questionnaire to analyze the choice architecture (qualitative study) were administered to each staff member.

In summer of 2020, a cross-sectional study was conducted in order to assess the impact of the nudge intervention in promoting vaccination uptake, and to describe the approach towards influenza vaccination during the COVID-19 pandemic. In July 2020, the survey was proposed to all the Tuscan NHs (about 300), and 111 agreed to participate, including the 8 NHs involved in the nudge intervention. Influenza vaccination uptake in 2018–2019 and 2019–2020 seasons, as well as the intention to get the vaccine in the 2020–2021 season, were investigated through an anonymous questionnaire (self-administered). As for the other studies, all the staff members were invited to join the survey by the Chief Officer.

### 2.2. The Nudge Intervention

Together with a questionnaire addressed to NH workers assessed to investigate the choice architecture in vaccination uptake, the accompanying cover letter offered the chance to shape an intervention to “nudge” the adoption of such behavior.

The cover letter was drawn up according to behavioral insight suggestions included in both the “non-participation form” contained in the 2019 WHO manual to promote vaccination uptake in healthcare workers [17], and the initiative funded by Public Health England to reduce antibiotic prescriptions among general practitioners in the United Kingdom [20].

The paper letter was personally addressed to every single NH worker, and signed by a high-profile figure (the Chief Director of the Tuscan Health Regional Agency and the Head of the Department of Health Sciences, University of Florence)—together with the NH Chief Officers—in order to increase the credibility of its content.

The content aimed to raise awareness not only on the professional responsibility towards fragile people in case of non-vaccination, but also on the personal working burden the NH workers would have had to deal with in case of colleagues getting the influenza virus.

The letter ends with the manifestation of trust by the NH Chief Officer in its personnel, and with the kind request to sign the delivering form and to compile the questionnaire in attachment.

Together with the questionnaire, a leaflet including all the useful information about how to get vaccinated was provided.

Differently from the WHO non-participation form, NH workers were not asked to sign the eventual denial of the vaccination uptake. The intervention aimed indeed to raise awareness on the risks of non-vaccination rather than make workers feel ‘threatened’ by social norms, since such feeling could have compromised the willingness to compile the questionnaire. For the same reason, the letter did not make use of social norms (comparisons among proper and improper behaviors) like in the intervention conducted in the United Kingdom.

### 2.3. Analysis of Choice Architecture: The Qualitative Study

#### 2.3.1. The Questionnaire

An anonymous paper-and pencil questionnaire was developed to detect the intentions to get the influenza vaccine in 2019–2020 season and the reasons for their choice (either for positive or negative intention). It consisted of three questions: the first, with closed answer (yes/not), to describe the intentions of getting the vaccination, the next two questions were open-ended and required a brief description of the reasons about the intention of getting the vaccination or not, respectively. Finally, there was the possibility to add comments. The questionnaire was administered to all the staff members of the 8 NHs involved in the study (*n* = 527), contextually with the nudge letter and the leaflet. Compilers put the fulfilled questionnaires in specific boxes, one for any NH. The administration and the collection of the questionnaires happened from 18th November to 10th December 2019.

#### 2.3.2. Qualitative Analysis on the Choice Architecture

Qualitative research is aimed to understand a phenomenon within a specific context, thoroughly exploring a single aspect, case, or issue. It has, therefore, a descriptive and explanatory function. Its goal is to understand the reasons underlying people’s behavior, deepening individuals’ point of view, their opinions, attitudes, and reference values: this approach was considered useful to deepen the personal motivations on NH staffs’ propensity to be vaccinated.

The analysis was carried out according to an inductive method, that is the construction of the theory formulated starting from the observation of the data collected. The analysis follows an iterative procedure, as the collection and analysis of the narrative material are in a circular relationship [21,22]. As the first step of the qualitative analysis, the research team read separately all the affirmative (yes group) and negative (no group) reasons about the intention to get the influenza vaccine reported by the respondents.

Within each group (yes group and no group), the research team identified broad and simple themes and categories, according to the cross-case logic (definition of categories/dimensions and search for similarities/differences within the groups) and with in-depth descriptions (thick description) and their labeling. Further analysis procedures were subsequently made to re-read the texts and redefine the initial categories (circular method), during which the starting categorical scheme was modified or specified. This process lasted until the research team felt they had achieved a satisfactory and complete coding scheme. After which, the relationships between themes were formulated, connecting the categories to each other in a coherent and unitary theory [21,22,23,24,25]. What emerged was compared with the so-called 5C model, a tool to categorize the psychological antecedents of vaccination and facilitate vaccination interventions’ design and evaluation.

This model identifies 5 factors: confidence (trust in the effectiveness and safety of vaccines, but also in the healthcare system and in the professionals who administer them), complacency (when the perceived risk, related to vaccine-preventable diseases, is low and therefore vaccination is not considered a necessary preventive action), constraints (the physical availability of vaccines, accessibility to services, and the ability to understand the problem affecting compliance with vaccination), calculation (the processing capacity and commitment of people in search for in-depth information: it is assumed that people with a high level of processing skills are more able to assess the risks of infections and vaccinations and therefore carry out a cost-benefit analysis), and collective responsibility (the willingness to protect others by vaccinating themselves, correlated with altruism. Its opposite is the willingness to rely on the protection derived from the vaccination of others, which is strictly connected with individualism) [26].

### 2.4. Cross-Sectional Study

From 15th July to 15th August 2020, a cross-sectional survey was conducted among Tuscan NHs, using a questionnaire almost completely comparable to that previously used in many different settings (NHs, hospital, university) [12,27,28,29]. It was administered on-line (the Chief Officer of each NHs shared the link to his staff) to collect individual data regarding influenza vaccination (self-reported) in 2018–2019 (yes/not), in 2019–2020 (yes/not), or intention to be vaccinated in 2020–2021 seasons (very likely, fairly likely, less likely, unlikely), knowledge, awareness, and attitudes concerning influenza and influenza vaccination (Likert-type), as well as demographic, educational, and health information. As far as health information is concerned, an assessment of self-perceived health status (from “1”-bad, to “10”-excellent) was collected. The staff members were also asked whether they live with children of less than 9 years, with elderly people, or with people with chronic diseases. Finally, the reasons for vaccination uptake or refusal in 2019–2020 were collected using closed-ended questions, selected according to what emerged in previous research [12,27,28,29] and to the results of the analysis of the choice architecture conducted in this study. The questionnaire had a NH identifier, but no individual identifiers to encourage completion.

As in the previous survey, a Vaccine Confidence Index (VCI) was calculated according to the literature [30], considering eight Likert-type statements included in the staff questionnaire to which the participants were asked to declare their agreement or disagreement. The statements were the following:

- Influenza is a serious illness (A1)
- Influenza vaccine is effective (A2)
- Healthcare workers must get vaccinated (A3)
- By getting vaccinated I protect people close to me from influenza (A4)
- It is better to get the flu than the vaccination (B1)
- Influenza vaccines have serious side effects (B2)
- Vaccine can cause influenza (B3)
- Opposed to vaccination (B4)

The level of agreement or disagreement was scored as follows: “totally agree” = 4, “partially agree” = 3, “partially disagree” = 2, “totally disagree” = 1. For the first four statements (A1–A4), the higher the Likert score, the better the propensity towards vaccines, while for the second four (B1–B4), the higher the Likert score, the lower the propensity.

The VCI was calculated as follows:

VCI = [ (A1 + A2 + A3 + A4)/4]/[(B1 + B2 + B3 + B4)/4](1)

where A1, A2, A3, and A4 were the scores to the first four statements, while B1, B2, B3, and B4 were those of the second four.

#### Statistical Analysis

Data on vaccination uptake and intention to vaccinate among the staff of the 8 NHs involved in the nudge intervention (hereinafter nudge group) were compared with those of the other 103 NHs that participated in the cross-sectional study (hereinafter comparison group). The same comparison was performed for the following variables: demographic, qualification, VCI score, living with children of less than 9 years, with elderly people, or with people with chronic diseases, and self-perceived health status. To compare vaccination uptake and the other categorical data, Fisher’s exact test or McNemar’s test were used, respectively for independent and paired samples. For continuous data, normality was assessed using the Kolmogorov-Smirnov test and then Student’s t test or the corresponding nonparametric tests were used. All the statistical analyses were performed using IBM SPSS 26, considering 0.05 as alfa level.

## 3. Results

### 3.1. Qualitative Analysis of Choice Architecture

As a whole, 40.2% (212/527) of the NH staff members fulfilled the questionnaire, and 51.8% of them (110/212) declared to be in favor of getting the influenza vaccination in the 2019–2020 season. Among those, 83.6% (92/110) expressed one or more reasons for this choice, while among the unfavorable, only 60.7% (62/102) stated the reasons.

#### 3.1.1. Reasons to Get Vaccinated

Three macro-categories emerged from the qualitative analysis of the answers that motivated the intention to get the vaccine; within each of them, sub-categories were then identified (Table 1).

A. Risk perception

The first identified macro-category refers to risk perception. It assumes an appropriate knowledge of the disease and the risks associated with it, the awareness of performing a profession in a context that exposes more to the likelihood of contracting the virus, and the desire to protect himself/herself and the others.

Among those workers who declared their intention in favor of getting the vaccine, 83.7% (77/92) have referred reasons in this macro-category, which therefore represents the core category of those who declared themselves in favor of vaccination. From the analysis of the motivations that fall within the perception of risk, two sub-categories were then identified: protection and awareness of working in a place at higher risk.

(A.1) Protection is an explicit intention aimed at protecting oneself and people nearby (family, colleagues, nursing homes residents, other people). In particular, most workers explicitly refer to the desire to protect both the private (themselves or their family) and the public dimension (colleagues and nursing home residents):

*“To avoid flu and to protect my family and NH residents, because they are fragile people” (respondent NH_5_3)*

*“In my opinion, vaccination is the (...) safest means of not transmitting it (influenza) to the NH residents and also to family members” (respondent NH_1-2_15)*

A smaller group is concerned only with personal protection and close affections:

*“I have already had the vaccine to prevent getting sick” (respondent NH_1-2_11)*

*“Protect me and my family” (respondent NH_1-2_21)*

Few refer to the protection of the public sphere alone:

*“To avoid infecting NH residents” (respondent NH_5_6)*

*“I would like to get vaccinated to protect the health of fragile subjects with whom I am in daily contact” (respondent NH_8_25)*

Although the main message in this subcategory is protection (towards himself/herself or others) through the vaccine, this motivation also contains an attitude of “trust in the vaccine”. This connection emerges mainly referring to personal protection or close relationships:

*“To raise my immunity” (respondent NH_8_64)*

*“Too many times I find myself with low immune defenses and therefore avoid or reduce the chances of catching other viruses, also protecting my family” (respondent NH 8_42)*

At the same time, the protection for colleagues and NHs residents, together with trust in the vaccine, calls for awareness of social responsibility, which is the will to protect others by vaccinating themselves:

*“Because I want to protect (...) especially all those who cannot be vaccinated” (respondent NH_1-2_34)*

*“Protection towards others and helping people without immune defenses who cannot be vaccinated to feel safe in the midst of many other people who have the possibility and the luck of being able to get vaccinated” (respondent NH_8_10)*

(A.2) Awareness of working in a place at higher risk. In this second sub-category, the awareness of being in a context that exposes more to the likelihood of contracting and transmitting influenza are mainly emphasized. Hence, the intention to adopt protective behaviors:

*“Since we do work at risk” (respondent NH_1-2_13)*

*“I work in a social health facility and there is a greater risk of getting the influenza” (respondent NH_8_18)*

B. Values Dimension

The second identified macro-category concerns the dimension of values, that is the set of ideals or norms of the individuals of a social group, which influence their action. Within this macro-category, two sub-categories have been identified.

(B.1) Prevention. The first sub-category refers to a generic motivation for prevention, meant as the will to prevent the negative effects of the influenza for individual and social benefit. This behavioral norm implies the appropriateness of a behavior aimed at preventing the disease. In this sense, although it is also closely linked to the perception of risk, prevention has been included in the dimension of values:

*“I think prevention is important” (respondent NH_8_73)*

(B.2) Social responsibility. The second sub-category refers to social responsibility, where the collective benefit stands out as a value that guides one’s intention to get vaccinated:

*“It is a favor that should not be wasted and a social duty” (respondent NH_5_3)*

*“Civic sense” (respondent NH_8_31)*

C. Trust

The third macro-category concerns trust, an attitude resulting from a positive assessment of facts, circumstances, relationships, for which one trusts-in this case-in the vaccine and more generally in the healthcare system and in the professionals, animated by a general feeling of safety. Trust can therefore be characterized into two sub-categories, as follow.

(C.1) Trust from experience/habits. It refers to the sense of safety and efficacy of the vaccine, mainly referred to the protection of one’s health. The basis of this choice is therefore the assumption that the vaccine works well because it is based on a positive experience, which corresponds to expectations:

*“In my opinion, vaccination is the most effective means of preventing influenza (...)” (respondent NH_1-2_15)*

*“I am in favor of vaccines. I don’t want to get sick” (respondent NH_6_3)*

This category also includes the motivations of staff members who refer to adopt the choice of getting vaccinated as a habit, recalling the systematic and repetitive nature of the action. Also, in this case, a reference to the expectations of the vaccine effectiveness is implicit, so much so that this choice becomes a personal habit:

*“Already done. Always done and I’m fine” (respondent NH_3_15)*

*“For many years I have been vaccinated to protect the health of assisted guests and my family members because until a few years ago I had a family member at risk” (respondent NH_6_4)*

As we can see from these motivations, trust in the vaccine recalls the macro-category of reference values (prevention and social responsibility) and awareness of the risk associated with contracting and transmitting influenza.

(C.2) Trust from the General Practitioner (GP). The trust coming from the GP or from other healthcare professionals includes the motivations of all staff members declaring to get the vaccine because they were advised by their referring doctor:

*“As an asthmatic, the family doctor strongly advised me to get the vaccine” (respondent NH_8_50)*

*“On the advice of doctors” (respondent NH_6_7)*

#### 3.1.2. Reasons to Not Get Vaccinated

Four macro-categories emerged from qualitative analysis of motivations for not being vaccinated; within each of them, sub-categories were then identified (Table 2).

A. Risk perception

As for motivations of those who declare their intention in favor of vaccine, the macro-category of risk perception is the most represented (55%) among those against the vaccine too. Their negative attitude is represented by poor or distorted knowledge of personal or social risk of influenza, assuming that it is not a serious disease. This option is strengthened by a lack of interest in exploring the topic.

From the analysis of answers related to risk perception, two sub-categories were then identified, “uselessness of vaccine” and “complacency”.

(A.1) Uselessness of vaccine. It is the most represented sub-category (42%) among those who are opposed to vaccination. It refers to the perception of unnecessariness about the opportunity of being vaccinated, with regard to one’s health.

Specifically, in the majority of cases, staff members motivated their perception of uselessness of vaccine with their “invulnerability” to seasonal flu, claiming that they never get sick:

*“It’s hard for me to get the influenza” (respondent NH_7_5)*

*“I am not a person who gets sick easily, my immune defenses are very strong” (respondent NH_1-2_18)*

It is worth noticing that this motivation contains a prominent attitude to “individualism”, a value referring to take care of oneself but not for others. It is absent the principle of reciprocity, which pushes an individual to get vaccinated to protect others, as others protect him/her.

Other respondents report a perception of “general uselessness”, without detailing why they believe that:

*“I believe it’s not necessary” (respondent NH_9_3)*

*“I think that getting vaccinated is not useful for me” (respondent NH_8_44)*

At last, in a few cases, uselessness is motivated by not being in contact with older people in the workplace. 

Compared to previous opinions, which mainly refer to generic or personal absence of risk perception, this motivation also reveals a lack of knowledge of flu ways of transmission:

*“I don’t work closely with residents and I don’t get sick frequently with influenza” (respondent NH_4_8)*

*“I am not in direct contact with patients” (respondent NH_8_6)*

(A.2) Complacency attitude. The perception of risk includes a second sub-category, the complacency attitude, that is affected by the habit. Here, the opposition to vaccination lies on maintaining the habit of never getting vaccinated.

In this case, the attitude seems to be oriented towards passivity, not questioning one’s choice, as well as towards a lack of values (already described) towards others’ protection and social responsibility. The habit of not getting vaccinated tends to affect the individual behavior much more than any negative experiences or distrust with vaccines. It is described as the prevailing power of inertia, and the maintenance of the status quo: changing one’s mind is in fact one of the most difficult challenges to fulfill for those who work on the architecture of choices in vaccine.

It is interesting to notice that habit has a double effect, on those who are in favor as well as on those against vaccination, but with opposite consequences: trust in personal and social benefit vs. disinterest for others and scarce risk perception for oneself:

*“I don’t get vaccinated because I never did it” (respondent NH_6_10)*

*“I haven’t been vaccinated until now, so I prefer not to do it this year either” (respondent NH_6_8)*

B. Distrust

The second macro-category refers to “distrust”, defined as an attitude of negative evaluation of facts, circumstances, and relationships, generating a feeling of distrust in others as well as in their own possibilities and generally producing a lack in self-confidence and peace of mind.

About the attitude concerning vaccination, in distrust, we can identify two sub-categories: distrust in safety and distrust in effectiveness.

“Distrust in effectiveness” is based on the belief that vaccination is an avoidable practice because it does not bring any benefits, even if it is not harmful, recalling the category of “uselessness of vaccine”.

(B.1) Distrust in safety. The majority of staff members who report reasons related to distrust refer to “distrust in safety” of vaccines, which seems to derive from negative experiences with vaccine:

*“I had physical weakness in previous times” (respondent NH_8_22)*

*“Because once I got vaccinated and I felt worse than when I didn’t” (respondent NH_5_19)*

(B.2) Distrust in effectiveness. The second sub-category, reported by a smaller number of workers, refers to “distrust in effectiveness” of vaccination practice:

*“Once I got vaccinated but I got the flu anyway” (respondent NH_1-2_2)*

*“I don’t think it’s effective” (respondent NH_1-2_3)*

C. Values Dimension

As for motivations of those who declare their intention in favor of vaccine, the macro-category of “values dimension” has been identified for those against as well. It includes a gap in ideals or norms based on healthcare workers’ awareness of their responsibilities, specifically on the norm of social protection (fragile and non-frail subjects), also achievable through personal protection. This can lead to opposition and criticism of the vaccine or to a partial perception of its individual and social usefulness.

Two sub-categories have been identified.

(C.1) Upset. Answers are characterized by short and poorly argued positions. In this case, respondents’ values seem to guide them towards a very extreme position of refusal to get vaccinated.

The transmitted message refers to the denial of flu vaccination importance:

*“Anti-vax” (respondent NH_9_1)*

*“I am against it” (respondent NH_8_60)*

(C.2) Lacking professional responsibility. The second sub-category refers to scarce or limited awareness of the responsibility of getting the vaccine owing to one’s profession. In general, this attitude calls for a lack of knowledge about the individual and social risk of contracting the flu virus. This does not necessarily involve totally disregarding vaccination importance, but it is not associated with a professional duty and it can lead to more extreme positions, i.e., the intention to “exploit” the others’ immunity for personal protection:

*“I take advantage of herd immunity of nursing home’s residents” (respondent NH_1-2_39)*

*“I personally believe that influenza vaccine is very useful for elderly and immunocompromised but not necessary for others, even those in contact with these diseases” (respondent NH_8_55)*

D. Reasons related to one’s health

The last macro-category identified refers to “reasons related to one’s health”.

Respondents justify their attitude towards vaccination as derived from aspects related to physical inability to do vaccines. Some workers, for example, declare that they cannot be vaccinated due to an autoimmune disease for which, indeed, this practice is not indicated:

*“I have an active autoimmune disease treated with immunosuppressive therapy” (respondent NH_8_1)*

*“Autoimmune subject. I do homeopathic vaccine” (respondent NH_7_13)*

However, in other cases, respondents’ health fears are not accredited by scientific evidence. This distrust or distorted knowledge tends to determine a negative attitude towards vaccination:

*“I have only one kidney and I don’t want to overload my organism” (respondent NH_6_1)*

*“This year I have already got other vaccinations and I don’t feel like getting too many vaccines in the same period (I probably can’t even get them close together)” (respondent NH_8_55)*

#### 3.1.3. Interpretative Model

**Figure 1:**
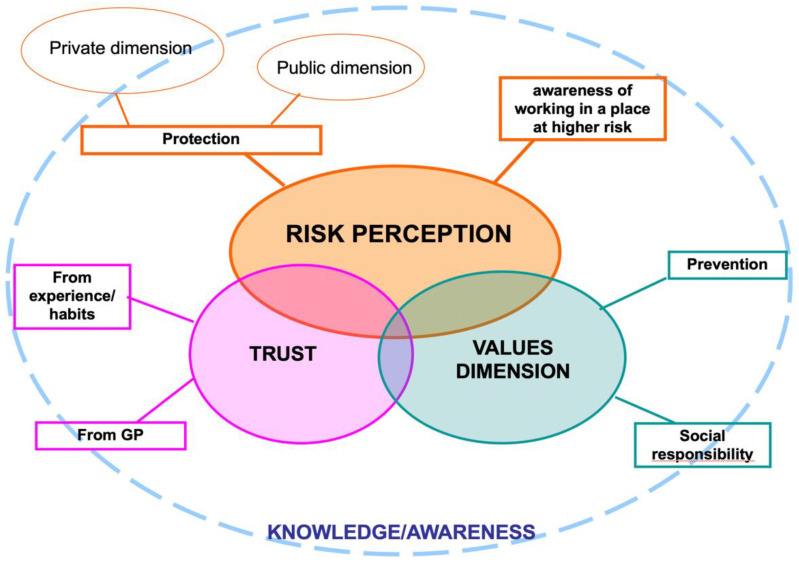
Links between macro-categories and sub-categories of reasons to get vaccinated.

**Figure 2:**
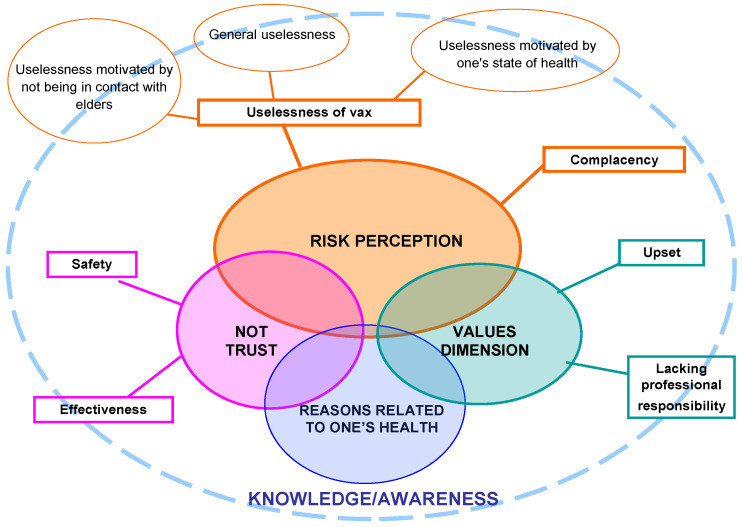
Links between macro-categories and sub-categories of reasons not to get vaccinated.

Figure 1 and Figure 2 show and summarize the logical connections, already described in the previous paragraphs, between the macro-categories and the related sub-categories identified as reasons that motivated the intention of getting or not getting the influenza vaccine by the NHs staff. In the figures, it is highlighted that the perception of risk is the core category of both groups of motivations.

In both Figure 1 and Figure 2, knowledge and awareness are in the background, although they have never emerged as explicit motivations of the respondents’ choices. A correct or distorted knowledge of the vaccination practice and the relative risks and benefits seems to be the basis of all the other macro-categories to which the various answers refer. Knowledge and awareness are connected with the perception of risk, with the correct interpretation of one’s health problems, with trust or mistrust towards the vaccine, and with the values dimension, on which attitudes and behaviors are based and from which we let ourselves be guided to make our decisions.

### 3.2. Data from the Cross-Sectional Study: The Nudge Group Versus the Comparison Group

As a whole, 2135 staff members fulfilled the questionnaire (compliance equal to 47.8%: 49% in the nudge group and 47.2% in the comparison group) but 90 were excluded due to excess of missing responses and 47 because it was not possible to identify the NH where the respondents worked. Then, the analyses were performed on 1998 questionnaires, with 195 included in the nudge group and 1803 in the comparison group, respectively.

Table 3 reports the results of the descriptive analysis, with the comparison of the two groups. Regarding demographic, health, and living condition data, the two groups do not significantly differ for any of the investigated variables, with the exception of “living with people with chronic diseases”: the percentage of staff members with this condition was significantly (*p* = 0.006) higher in the comparison group (17.3%) than in the nudge group (8.7%). Influenza vaccination uptake in the 2018–2019 season was similar in the two groups (23.6% vs. 22.2% respectively, in the nudge and comparison group; *p* = 0.35), but it was significantly different in the 2019–2020 season (*p* = 0.006): 28% in the nudge group (+4.4% with respect to 2018–2019) and 20% in the comparison group (−2.1% with respect to 2018–2019). Also, the intention to get the vaccine in the 2020–2021 season was significantly different in the two groups (*p* = 0.027): in the nudge group, 37.9% of the staff members stated it was very likely they will get the vaccine in the 2020–2021 season, while in the comparison group, that percentage was equal to 30.8%.

Reasons for vaccination uptake or not in the 2019–2020 season are described in Table 4. Concerning the effectiveness of the vaccine (distrust in effectiveness), which consequently caused not having been vaccinated (“the vaccine does not work”), responses were significantly higher in the comparison group than the nudge group (16.2% vs. 9.3%). For the other listed reasons, either regarding vaccination uptake or not, no significant differences were observed between groups. Nonetheless, among vaccinated staff members, reasons attributable to personal motivations (prevention and trust form the experience: i.e., “I do not want to get sick”, “I am vaccinated every year”, “I was sick with influenza in the past”) or to the vaccination uptake of close people (trust form the experience: “my colleagues or relatives get the vaccine”) tended to be more frequently reported in the comparison group. On the other hand, in the nudge group, statements such as “I have been recommended vaccination” and “I felt compelled to be vaccinated” (trust in vaccination) tended to be more frequently reported as motivations for getting the vaccine. Considering not vaccinated staff members, the consolidated habits of not getting the vaccination (complacency attitude: “I have never been vaccinated before”) tended to be more frequently reported in the nudge group as motivation for not having received the vaccine, as well as the absence of personal experience with the disease (uselessness of vaccine: “I never get sick with influenza”). In the comparison group, responses concerning the vaccine such as “fear of side effects”, “I am concerned about getting influenza from the vaccine” (distrust in safety), “I am not in the target group” (risk perception), and “I did not have time to be vaccinated” tended to be more frequently reported as motivations for not having received the influenza vaccination in 2019–2020.

## 4. Discussion

Vaccination is a preventive and social practice strongly based on the behavior of individuals, in case of no obligation by law. Accordingly, behavioral science can be useful in understanding the reasons for vaccination acceptance or refusal, as well as to promote its spread [18,31,32]. For this reason, we performed the present study with the aim of understanding the choice architecture of influenza vaccination acceptance or refusal among the staff of NHs and to promote vaccination acceptance using the nudge approach.

### 4.1. Choice Architecture

The qualitative analysis of the reasons for the intention of getting or not getting the influenza vaccination has shown some cognitive biases in making this decision, as well as positive attitudes, according to the literature [31].

Regarding the intention to get the vaccine, social responsibility, that is the desire to protect others (colleagues and NH residents) by getting vaccinated, seemed to be a key issue. The principle of reciprocity can be considered as the base of this reason, as also described by the World Health Organization [33]: people are more willing to be vaccinated if they know that—by means of vaccination—they can protect the others and the others can protect themselves. In literature, this attitude is included among the strategic behaviors of “*altruism*”. The opposite principle is “*parasitism*”, explicitly expressed by one participant as the reason not to be vaccinated: it is the attitude to protect oneself by leveraging the protection of others (herd immunity) [34]. Parasitism can be linked to a lack of professional responsibility. Healthcare professionals should share the value of social protection as a professional responsibility, not just as a duty towards vulnerable individuals. The study reveals a gap in this regard as well as a limited perception of the risk to one’s health. These workers, who motivate the answer with their “invulnerability” to seasonal influenza, also show a gap in professional ethics: the sense of common good, which includes the respect for rules, laws, and attitudes towards people and colleagues, is fundamental for the sense of taking care of residents of health and social facilities. Jonas underlines the importance of the ethics of responsibility and the ethics of care, linking both of them to the fear of the patient’s vulnerability. The ethics of care can be considered a way to counteract current indifference [35].

Lacking professional responsibility, in addition to a lack of knowledge upon the risks given by influenza, also emerges among those who did not want to get vaccinated because they were not in direct contact with the NH residents. In this regard, WHO reports that NH staff, independently from the job role, should be vaccinated because they could represent a source of infection for their colleagues [17].

Additionally, another common determinant of non-vaccination is the belief that vaccines are unsafe [36], and/or associated with serious diseases and unknown long-term side effects. This distrust is also recalled by those who fear the side effects of the vaccine on their current health problems, even when evidence does not support this relationship.

The factors that determine the staff’s intention or not to get vaccinated are in line with the 5C model by Betsch et al. [26]. The model has been developed as a lean tool to categorize the determinants of vaccination, monitor the hesitancy to get vaccinated, and facilitate the planning and evaluation of vaccination implementation interventions. It comprises five main categories: confidence, complacency, constraints, calculation, and collective responsibility.

From our analysis, three factors clearly emerge referring to the 5C Model, both in favor of and against vaccination: confidence, complacency, and collective responsibility (the willingness to protect others by vaccinating themselves, correlated with altruism. Its opposite is the willingness to rely on the protection derived from the vaccination of others, which is strictly connected with individualism). With regard to the calculation, more in-depth analyses would be required. On the other hand, the constraints category does not emerge as an obstacle or facilitator. Since the constraint issue emerged as one of the reasons for not-getting vaccinated in the cross-sectional study (“I did not have time”), we can suppose that physical barriers, such as difficulties in accessibility to the services, could contribute to explaining the differences between the intention to get the vaccine and the vaccination uptake.

### 4.2. The Nudge Intervention in Promoting Influenza Vaccination Uptake

According to the results, a nudge intervention like that applied in this study-i.e., a personal-addressed paper letter, signed by high-profile figures and aimed to raise awareness about the professional responsibility towards fragile people and colleagues, delivered with an information leaflet—could be useful in promoting influenza vaccination uptake. In fact, after the nudge intervention, both influenza vaccination uptake in the 2019–2020 season and intention of getting the vaccine in the 2020–2021 season were significantly higher in the nudge group than in the comparison group, while in the previous season (2018–2019), vaccination uptake was similar in the two groups. Moreover, respondents of the two groups were similar with respect to the Vaccine Confidence Index, that has been indicated as the major predictor of influenza vaccination acceptance among Tuscan NHs staff in a previous study [12]. The effect of the nudge intervention is supported by behavioral science theories and seems to be confirmed also by analyzing the reported motivations for vaccine uptake or non-uptake, which tend to be different in the nudge group with respect to the comparison group (although without statistically significant differences).

Nudging can be considered as a theoretically grounded, potentially effective way to address the behavior gap in promoting healthy habits, such has healthy eating, as well as in designing and implementing public health policies [32,37,38]. It is becoming more and more popular since it offers a cheap and easy-to-perform tool by offering a guidance, without enforcement, on individual behavioral change that is good for and, on reflection, preferred by, individuals themselves [39]. To introduce a nudge approach in this study, a “nudging” cover letter, according to the form suggested by the WHO manual [19], was drawn up and administered together with the questionnaire to investigate NH workers’ choice architecture about vaccination uptake. In line with the literature, our nudge intervention had no other cost than the few hours the team spent to simply discuss and implement the behaviorally informed document.

Just a few primary studies have been already conducted in assessing the effectiveness of nudging in promoting vaccination uptake. Some of them have described the results of nudge interventions aimed at increasing influenza vaccination uptake—although different from those that have been used in this study—with inconsistent results: some authors have described a significant increase in vaccination acceptance, while others found no effects [40,41,42].

Moreover, to the best of our knowledge, this is the first research conducted among NH staff, including both healthcare and non-healthcare workers, with no possible international comparisons for the same target. Future studies should be realized in order to confirm the effectiveness of nudging in promoting influenza vaccination uptake among NH staff, and to assess whether different nudge interventions lead to different results.

### 4.3. Limitations of the Study

This study has some limitations. First of all, the participation in the qualitative study and in the nudge intervention was voluntary, so that the sample has to be considered as one of convenience. In particular, a selection bias, either at the NH or at the individual level, could be present. The Chief Officers who agreed to participate should have been those with more interest in promoting influenza vaccination uptake in the NHs they direct. Moreover, also the staff members who responded to the questionnaire for the analysis of the choice architecture should have been those more interested in sharing their opinion of influenza vaccination. Furthermore, in some NHs, the percentage of the staff that fulfilled the questionnaire was quite low, and since it was not possible to investigate the reasons for low compliance in some facilities, this aspect could have introduced another selection bias. Similar limits can be listed for the cross-sectional survey as well, although a large number of NHs participated. Additionally, many Chief Officers agreed to participate in the cross-sectional study, so that the two groups-the nudge group and the comparison group-are numerically unbalanced. This aspect could have affected the comparison.

Overall, these aspects lead to limitations in the generalizability of the results.

Regarding collected variables, the recall bias could have influenced data on vaccination uptake in the 2018–2019 season, while the social desirability bias could have influenced some of the expressions of motivations for vaccination uptake or not. Regarding the latter, anonymity should have limited the bias.

Finally, it is to note that the cross-sectional study was conducted during the COVID-19 pandemic, in particular at the end of the first wave in Italy. The effect of the pandemic is supposed to be the same on the participants of both the nudge and the comparison group, so it can be supposed not to be a confounding factor when comparing data regarding the two groups. On the other hand, it could have affected the intention to get vaccinated in the 2020–2021 season, that actually resulted quite high in both groups.

## 5. Conclusions

Although the results of this study need to be confirmed in future, larger studies, we can conclude that nudge interventions-that are simple, fast, and low cost-could be effective in promoting vaccination acceptance among NH staff and the analysis of choice architecture could be useful in improving tailored, new nudge interventions aimed at changing irrational biased and cognitive errors.

In fact, in the case of vaccinations, as well as for many other decisions in everyday life, people are conditioned to too much conflicting and opposing information, independently from the real “truth” or scientific evidence. This is the reason why, also in the case of being vaccinated, people who should be favorable to being vaccinated-as is the case of healthcare professionals-are often hesitant and need a sort of gentle push, as nudge has been defined.

## Figures and Tables

**Table 1:** Macro-categories and subcategories of reasons to get vaccinated.

Macro-Category	Description	Sub-Categories
Risk Perception	Appropriate knowledge of the disease and its risks, awareness of working in a place at higher risk of contagion, willingness to protect oneself and others	Protection: protection for oneself and his/her net of contacts
		Awareness of working in a place at higher risk: awareness of being in a context that exposes more to the likelihood of contracting and transmitting influenza are mainly emphasized
Values Dimension	The set of ideals or norms of the individuals of a social group, which influence their action	Prevention: the will to prevent the negative effects of the influenza for individual and social benefit
		Social Responsibility: the collective benefit stands out as a value that guides one’s intention to get vaccinated
Trust	An attitude resulting from a positive assessment of facts, circumstances, relationships, for which one trusts in the vaccine and more generally in the healthcare system and in the professionals, animated by a general feeling of safety	From experience/habits: the sense of safety and efficacy of the vaccine, mainly referred to the protection of one’s health
		From General Practitioner (GP): safety and efficacy of the vaccine from GP or healthcare professionals

**Table 2:** Macro-categories and subcategories to not get vaccinated.

Macro-Category	Description	Sub-Categories
Risk Perception	Poor or distorted knowledge of personal or social risk related to flu assuming that there is no danger. This option is strengthened by a lack of interest in exploring the topic	Uselessness of vaccine: perception of unnecessariness about the opportunity of being vaccinated, with regard to one’s health
		Complacency: attitude oriented towards passivity, not questioning one’s choice, lack of values towards others’ protection and social responsibility
Distrust	Attitude to negative evaluation of facts, circumstances, and relationships generating a feeling of distrust in others as well as in their own possibilities and generally producing a lack of self-confidence and peace of mind	Distrust in safety: belief that vaccines can be associated to serious diseases, collateral and unknown long-term effects, and belief that risks are greater than benefits
		Distrust in effectiveness: belief that vaccination is an avoidable practice because it does not bring any benefits, even if it is not harmful
Values Dimension	Gaps in ideals or norms based on the awareness of the healthcare workers’ responsibilities, specifically on the norm of social protection (fragile and non-frail subjects), also achievable through personal protection. This can lead to opposition and criticism of the vaccine or partial perception of its individual and social usefulness.	Upset: very extreme position of refusal to get vaccinated
		Lacking professional responsibility: refers to scarce or limited awareness of the responsibility of getting vaccinated due to the kind of profession
Reasons related to one’s health	Attitude towards vaccination as derived from aspects related to physical inability to be vaccinated	-

**Table 3:** Descriptive analysis of the collected variables by group. VCI: Vaccine Confidence Index, SD: standard deviation.

Categorical Variables		Whole Sample (*n* = 1998)	Nudge Group (*n* = 195)	Comparison Group (*n* = 1803)	*p* *
		%	%	%	
Females		86.9	87.2	86.9	0.879
Native speakers-Italian		84.9	87.2	84.6	0.981
Educational level	Higher than bachelor’s degree	2.6	2.6	2.6	0.088
	Bachelor’s degree	18.7	17.4	18.9	
	High school degree	46.5	55.4	45.6	
	Less than high school diploma	31.2	23.1	33	
Qualification	Nurses	12.8	11.8	12.9	0.251
	Physiotherapists	4.3	4.6	4.2	
	Health educators	4	3.6	4	
	Assistants/aides	58.8	55.4	59.1	
	Cleaning staff	5.9	10.3	5.4	
	Other nonclinical staff	6.4	7.7	6.3	
	Other clinical staff	6.6	5.1	6.8	
Living with children of less than 9 years		19.6	17.9	19.8	0.604
Living with elderly people		20.1	15.4	20.6	0.22
Living with people with chronic diseases		16.5	8.7	17.3	0.006
Vaccination uptake 2018–2019		22.3	23.6	22.1	0.35
Vaccination uptake 2019–2020		20.8	28	20	0.006
Intention to vaccinate in 2020–2021 (very likely)		31.5	37.9	30.8	0.027
**Numerical Variables**		**Mean ± SD; Median**	**Mean ± SD; Median**	**Mean ± SD; Median**	***p* °**
Age ^#^		44.4 ± 11.1; 46	44.6 ± 10.9; 47	44.3 ± 11.1; 46	0.791
VCI ^#^		1.61 ± 0.83; 1.3	1.67 ± 0.86; 1.3	1.60 ± 0.83; 1.33	0.358
Health status ^#^		8.5 ± 1.3; 9.0	8.5 ± 1.3; 9	8.5 ± 1.3; 9.0	0.515

* Fisher’s exact test (Nudge vs. Comparison group); ° Mann-Whitney test (Nudge vs. Comparison group); ^#^ Kolmogorov-Smirnov test: *p* < 0.01.

**Table 4:** Motivation for vaccination uptake or non-uptake in the 2019–2020 season, by group.

Motivation for Vaccination Uptake in 2019–2020	Nudge Group (*n* = 55)	Comparison Group (*n* = 360)	*p* (Fisher’s Exact Test)
I do not want to get sick	76.4	79.7	0.339
Concerning my job, it is important to protect people in contact with me	85.5	84.7	1
I am vaccinated every year	58.2	66.9	0.223
I was sick with influenza in the past	30.9	42.5	0.108
The vaccine administration was convenient	50.9	48.1	0.772
I have been advised to get vaccinated	63.6	54.4	0.224
My colleagues or relatives get the vaccine	20	26.1	0.406
I felt compelled to be vaccinated	78.2	71.7	0.337
**Motivation for Vaccination NON-Uptake in 2019–2020**	**Nudge Group (*n* = 140)**	**Comparison Group (*n* = 1443)**	***p* (Fisher’s Exact test)**
I am not in the target group	34.3	41.3	0.125
Fear of side effects	17.9	24.8	0.078
The vaccine does not work	9.3	16.2	0.028
I am concerned about getting influenza from the vaccine	12.1	15.2	0.385
I never get sick with influenza	47.1	40.3	0.126
The vaccine administration was not convenient	3.6	5.3	0.547
I did not have time to be vaccinated	4.3	8.5	0.103
I forgot to be vaccinated	4.3	6.2	0.458
Fear of needles	5.7	6.3	1
No one informed me about the vaccination campaign	4.3	6.6	0.366
I have never been vaccinated before	45.7	38.1	0.084
I did not think about it	19.3	19.3	1
